# Avoiding Post-DMEK IOP Elevation: Insights from a Standardized Surgical Approach

**DOI:** 10.3390/jcm15020521

**Published:** 2026-01-08

**Authors:** Stephanie D. Grabitz, Anna L. Engel, Mohammad Al Hariri, Adrian Gericke, Norbert Pfeiffer, Joanna Wasielica-Poslednik

**Affiliations:** Department of Ophthalmology, University Medical Center of the Johannes Gutenberg University of Mainz, Langenbeckstr. 1, 55131 Mainz, Germany; stephanie.grabitz@unimedizin-mainz.de (S.D.G.); annalarissa.engel@unimedizin-mainz.de (A.L.E.); mohammad.alhariri@unimedizin-mainz.de (M.A.H.); adrian.gericke@unimedizin-mainz.de (A.G.); norbert.pfeiffer@unimeidzin-mainz.de (N.P.)

**Keywords:** keratoplasty, Descemet membrane endothelial keratoplasty, DMEK, pupillary block, eye pressure elevation after DMEK, venting, Ando iridectomy

## Abstract

**Background:** Descemet membrane endothelial keratoplasty (DMEK) is the most frequently performed keratoplasty procedure in many countries. One of the most common early complications is an elevation of intraocular pressure (IOP). The aim of this study was to characterize early postoperative IOP behavior following DMEK performed with 10% sulfur hexafluoride (SF_6_) tamponade and to determine the frequency and timing of required IOP-lowering interventions within the first 48 h. **Methods:** We retrospectively reviewed postoperative outcomes of 116 consecutive DMEK procedures between May and December 2024 at the University Medical Center in Mainz, Germany. No specific exclusion criteria were applied. All surgeries included a surgical iridectomy at the 6 o’clock position, 10% (SF_6_) tamponade, and maintaining a mid-normal IOP at the end of surgery. Postoperative assessments included IOP measured using Goldmann applanation tonometry, the percentage of gas fill in the anterior chamber evaluated at the slit lamp, and the need for IOP-lowering interventions as determined by the on-call resident at 3, 24, and 48 h after surgery. IOP-lowering interventions consisted of venting in cases of elevated IOP, gas fill > 90%, and/or suspected angle closure or pupillary block, as well as intravenous or oral acetazolamide in cases of moderate IOP elevation with a lower gas fill and a patent iridectomy. If a single intervention was insufficient, a combined approach was used. **Results:** A total of 116 eyes from 98 patients (62 female, mean age 73.0 ± 9.8 years) were analyzed. DMEK was combined with cataract surgery in 41 eyes, and 4 eyes underwent phakic DMEK. Postoperatively, all iridectomies remained patent, and no cases of pupillary block occurred. Mean IOP and gas fill were within normal limits and declined steadily during the first 48 h. IOP-lowering procedures were performed in 11 eyes (9.5%), including venting (*n* = 3), acetazolamide administration (*n* = 7), and a combination of both (*n* = 1). There was no difference between DMEK and triple-DMEK regarding postoperative gas fill, IOP, or the need for IOP-lowering interventions. Mean postoperative IOP was significantly higher, and IOP-lowering interventions were more frequent in glaucoma vs. non-glaucoma patients. Re-bubbling was performed in 12 eyes (10.3%). Two cases of primary graft failure (1.7%) were recorded. **Conclusions:** In our patient cohort, a standardized surgical approach incorporating a surgical iridectomy at the 6 o’clock position, 10% SF_6_ tamponade, and maintaining a mid-normal IOP at the end of surgery effectively prevented pupillary block. We recommend early postoperative assessment of IOP and percent gas fill to promptly identify and manage impending IOP elevation, which is particularly important in patients with glaucoma.

## 1. Introduction

Lamellar keratoplasty has revolutionized corneal transplantation by offering a selective, tissue-sparing approach to restore vision. Among its techniques, Descemet membrane endothelial keratoplasty (DMEK), introduced by Melles et al. in 2006, has emerged as the gold standard for treating endothelial dysfunction while preserving overall corneal architecture [1]. Over the past decade, DMEK has gained widespread acceptance and now accounts for approximately 70% of all keratoplasties and 98% of posterior lamellar keratoplasties in Germany, contributing to a doubling of the annual number of corneal transplants performed nationwide [2].

Despite its clinical advantages, including faster visual recovery, lower rejection rates, and improved optical outcomes, DMEK also carries procedure-specific risks. Early postoperative elevation of intraocular pressure (IOP) is among the most significant complications, as untreated pressure spikes may compromise graft survival and lead to iris or optic nerve damage. Previous studies have reported early IOP elevations in roughly 10–20% of DMEK patients [3,4,5]. Elevated IOP has been associated with substantial endothelial cell loss in both experimental and clinical studies, with reports of up to 33% in cases where IOP reached 55 mmHg [6]. Xie et al. demonstrated that intermediate-term IOP elevation after DMEK may reduce graft endothelial cell density and lead to graft failure within six months after surgery [7]. Pupillary block represents the most feared cause of acute postoperative IOP elevation, typically resulting from mechanical obstruction of aqueous outflow by the air or gas tamponade used to secure the graft [8]. To mitigate this risk, most surgical protocols include a prophylactic peripheral iridotomy or surgical iridectomy, usually at the six o’clock position [9]. Nevertheless, postoperative IOP elevation may still occur due to other factors such as overfilling of the anterior chamber, impaired aqueous outflow, preexisting glaucoma, or the use of higher concentrations of gas tamponade [10]. In these cases, timely intervention—such as decompression via venting, pharmacologic IOP reduction, or revision of the iridotomy or iridectomy—is critical.

The type and concentration of the anterior chamber tamponade may influence the risk of postoperative IOP elevation. Previous studies comparing air, 10% sulfur hexafluoride (SF_6_), and 20% SF_6_ have mainly concentrated on graft detachment and re-bubbling rates. While 20% SF_6_ has shown a lower rate of graft detachment, its longer intraocular persistence may carry a higher risk of IOP elevation [11,12]. In contrast, 10% SF_6_ provides a shorter-acting tamponade, offering potentially greater safety with respect to postoperative IOP elevations [13]. However, evidence on early IOP behavior specifically following DMEK with 10% SF_6_ is limited. Furthermore, data on the earliest postoperative time points are scarce. Most studies report IOP at 24 h or later, even though clinically significant IOP elevations and pupillary block may occur within the first few hours after surgery.

At our institution, a 3 h postoperative assessment is routinely performed to detect and manage potentially sight-threatening IOP elevations at an early stage. Despite its clinical relevance, the effectiveness and necessity of this very early follow-up have not been systematically evaluated. Therefore, the aim of this study was to characterize early postoperative IOP behavior following DMEK performed with 10% SF_6_ tamponade, to determine the frequency and timing of required IOP-lowering interventions within the first 48 h, and to compare outcomes between glaucoma and non-glaucoma patients. By focusing on very early postoperative time points, this study addresses an important knowledge gap and provides clinically relevant insight into the immediate postoperative course following DMEK.

## 2. Material and Methods

We retrospectively reviewed and analyzed postoperative outcomes of all consecutive patients, who underwent DMEK or triple DMEK between May and December 2024 at the University Medical Center in Mainz, Germany. No specific exclusion criteria were applied. According to local law (‘Landeskrankenhausgesetz’ §36, §37), no ethical approval was required for this retrospective review.

### 2.1. Eye Banking and DMEK Graft Preparation

Eye banking and DMEK graft preparation was performed at our institution’s standard of care [14]. In brief, the donor corneas were obtained and stored in the Eye Bank of Rhineland-Palatinate in Mainz. All donor corneas were stored in Dextran-free medium 1 (Cat-No. F-9016; Biochrom, Berlin, Germany) at 34° Celsius and were transferred into dextran-containing medium 2 (Cat-No. F-9017; Biochrom) 24 h prior to graft preparation. Medium 2 is supplemented with 60 g Dextran 500 per 1000 mL as opposed to medium 1. Both media 1 and 2 were supplemented with gamma-irradiated fetal calf serum 10% (No. S0415, Biochrom).

The Descemet endothelial complexes (DEC) were prepared using the stripping technique on the day before or on the same day as DMEK by two experienced surgeons. To avoid an upside-down orientation all grafts were marked with a technique described previously [15].

### 2.2. DMEK Surgery

DMEK procedures were performed under general or topical anesthesia by two experienced corneal surgeons. All surgeries included a surgical peripheral iridectomy at the 6 o’clock position and tamponade with a standardized mixture of 90% air and 10% SF_6_. Most of the Descemet endothelial complexes (DECs) had a diameter of 8.0–8.5 mm. The descemetorhexis was performed within an area of 8.0–8.5 mm depending on the size of the graft using an irrigation Descemet hook (G-38601, Geuder AG, Heidelberg, Germany) and an irrigation Descemet scraper (G38602, Geuder AG, Heidelberg, Germany). The single-use DMEK-cartridge (G-38635, Geuder AG, Heidelberg, Germany) was used to insert the graft into the anterior chamber. The graft was unfolded by gentle tapping of the cornea. The positioning of the graft was checked by the orientation of the mark, which was visible in all cases. In case of an upside-down positioning, the graft was flipped over with a flush of balanced salt solution, and the position was checked again. An 90% air/10% SF_6_ bubble was injected underneath the graft as soon as the proper orientation and centration was achieved. The paracenteses were not sutured.

At the end of surgery, the palpably assessed IOP showed mid-normal values, and anterior chamber was not overfilled with air/gas. All patients were instructed to remain in a strict supine position for three hours postoperatively and more loosely for several days until gas resorption.

If DMEK was combined with cataract surgery (triple-DMEK), phacoemulsification and implantation of a monofocal hydrophobic intraocular lens were performed first. After pharmacological pupil constriction with acetylcholine, the DMEK procedure was carried out as described above.

### 2.3. Postoperative Evaluation and IOP-Lowering Interventions

**Figure 1:**
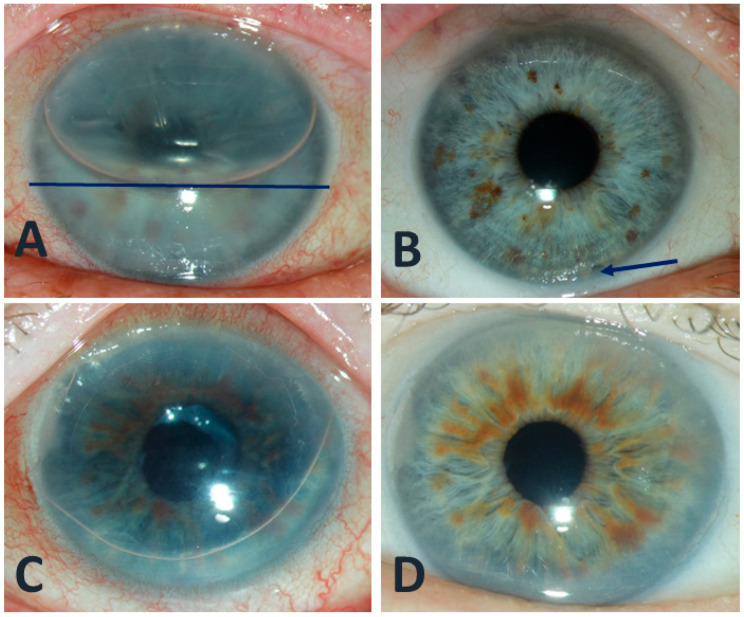
(**A**) Three-hour-follow-up after DMEK with normal IOP, gas fill of ≈60% (horizontal blue line) and transient epithelial edema in a timely manner. (**B**) Clear cornea 3 weeks after DMEK, patent iridectomy at 5:30 o’clock position (blue arrow). (**C**) Three-hour follow-up after DMEK showing a pupillary block and elevated IOP due to the absence of an iridectomy, treated with venting at this early postoperative time point. (**D**) Clear cornea from image C, two weeks after DMEK and venting.

The initial postoperative evaluation was performed three hours after surgery and included a slit-lamp examination to estimate gas fill, rule out pupillary block, and confirm iridectomy patency. IOP was measured using Goldmann applanation tonometry (Haag-Streit, Koeniz, Switzerland). Further IOP and gas fill assessments were conducted at 24 and 48 h postoperatively (Figure 1). The decision to perform IOP-lowering interventions at the 3 h-follow-up was made by the on-call ophthalmology resident, based on clinical judgment and IOP readings.

Possible IOP-lowering interventions included the following. Venting, which involves opening one of the paracenteses to reduce gas fill, was performed in cases with elevated IOP (no specific values were defined), and/or gas fill greater than 90%, and/or suspected angle closure or pupillary block. Intravenous or oral acetazolamide was administered in cases of moderate IOP elevation (individual decision making depending on IOP level, presence of glaucoma, patients’ symptoms) with a gas fill lower than 90% and a patent iridectomy. In situations where the response to a single intervention was insufficient, a combined approach was employed.

In general an IOP > 21 mmHg at any postoperative visit was considered as clinically significant. For patients with advanced glaucoma and a lower individual target IOP was considered. As this was a real-life observation rather than a prospective study protocol, decisions regarding IOP-lowering interventions were subjective and based on the individual experience of the residents.

In Table 1, we describe in detail all 11 cases requiring IOP-lowering interventions to illustrate the clinical situations and the corresponding decisions made.

### 2.4. Outcomes

Primary outcomes included the incidence and type of IOP-lowering intervention, changes in IOP and anterior chamber gas fill over time, and postoperative complications such as re-bubbling or graft failure. Subgroup analyses compared:

DMEK-only vs. triple-DMEK and eyes with and without preexisting glaucoma

### 2.5. Power Statement and Statistical Analysis

We prospectively conducted a power calculation in the study cohort prior to the start of data analysis. Assuming a two-tailed alpha of 0.05, using a t-test comparing two means from paired samples (n = 116) comparing postoperative IOP- and gas fill-levels at different time points. With an observed first postoperative IOP of 17 mmHg and gas fill of 65%, respectively, the current study yielded a power of >0.95 to detect a change of more than 2 mmHg in IOP and a change of more than 5% in gas fill, respectively.

Data management and statistical analyses were performed in Stata software, version 16.0 (Stata Corp LP, College Station, TX, USA). Quantitative values are expressed as mean ± standard deviation (SD). Student’s *t*-tests (normally distributed variables), Mann–Whitney-U Test (skewed distribution) or chi-square (binary variables) were used for comparisons as appropriate. A two-tailed *p* value of <0.05 was considered significant. Due to the explorative nature of this analysis no correction for multiple testing was applied increasing the risk of Type 1 errors.

## 3. Results

A total of 116 eyes from 98 patients were included in the study. The mean age of the patients was 73.0 ± 9.8 years, and 62 of the patients were female. The most common indication for DMEK was Fuchs’ endothelial corneal dystrophy, accounting for 102 eyes. Additional indications included pseudophakic bullous keratopathy in seven eyes, past herpes simplex virus-related endotheliitis in two eyes, uveitis in one eye, iridocorneal endothelial syndrome in one eye, previous penetrating keratoplasty in one eye, and a history of complicated glaucoma surgery in two eyes.

DMEK was performed in combination with cataract surgery (triple-DMEK) in 41 eyes, while four eyes underwent phakic DMEK without lens removal. In all other cases the eyes were pseudophakic.

All procedures were uneventful, with no cases of upside-down DEC positioning based on the orientation mark. The peripheral iridectomy at the 6 o’clock position remained patent in all eyes, and no pupillary block or angle closure was observed postoperatively

### 3.1. Postoperative IOP and Gas Fill

**Figure 2:**
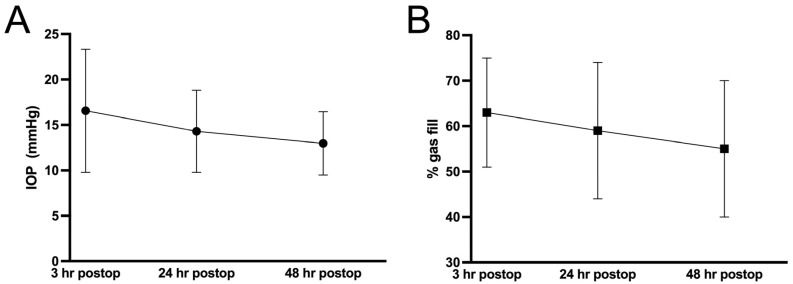
Changes in mean intraocular pressure (IOP; (**A**)) and mean anterior chamber gas fill (**B**) during the first 48 h postoperatively. Error bars represent the standard deviation.

Mean IOP and anterior chamber gas fill were recorded at three time points postoperatively. At 3 h, the mean IOP was 16.6 ± 6.8 mmHg, and the mean gas fill was 63 ± 12%. At 24 h, the mean IOP had decreased to 14.3 ± 4.5 mmHg, and gas fill was 59 ± 15%. At 48 h, the mean IOP further declined to 13.0 ± 3.5 mmHg, and gas fill was measured at 55 ± 15% (Figure 2).

### 3.2. IOP-Lowering Interventions

Intraocular pressure-lowering interventions were performed in 11 eyes, representing 9.5% of the total cohort. Among these, venting was performed in three eyes (#1 to #3) systemic acetazolamide was administered in seven eyes (#5 to #11), and one eye received both treatments (#4). Characteristics of the eyes that received IOP-lowering interventions, as well as the corresponding time points (3 or 24 h postoperatively), are presented in Table 1. Four of the 11 eyes that required intervention had a known history of glaucoma or glaucoma surgery. No complications were reported in association with the IOP-lowering interventions.

Re-bubbling was necessary in twelve eyes (10.3%), including one with a previous venting maneuver (#1). Two cases of primary graft failure were recorded during the postoperative period (1.7%). No urgent surgical re-intervention was required, and all other cases proceeded without severe complications.

### 3.3. DMEK vs. Triple-DMEK

The mean preoperative IOP was 13.3 ± 2.1 in DMEK patients and 14.3 ± 2.8 mmHg in Triple-DMEK patients. Mean age was 75 ± 9.9 years in the DMEK patients and 69.7, years in the Triple-DMEK group. Graft size was not different between glaucoma and non-glaucoma groups. All surgeries were performed by two experienced surgeons (JWP and MA), with a distribution of 83.6% versus 16.4% in the DMEK group and 100% versus 0% in the Triple-DMEK group.

We did not observe any significant differences between patients, who underwent DMEK alone (n = 74) and those who received triple procedure (phacoemulsification, lens implantation and DMEK) (n = 41) in terms of postoperative gas fill, IOP, or the need for IOP-lowering interventions. Mean gas fill at 3, 24, and 48 h postoperatively was nearly identical between groups: at 3 h, 63 ± 0.14% (DMEK) vs. 63 ± 0.09% (triple-DMEK), *p* = 0.99; at 24 h, 59 ± 0.17% vs. 58 ± 0.11%, *p* = 0.67; and at 48 h, 55 ± 0.17% vs. 56 ± 0.12%, *p* = 0.60. IOP values followed a similar trend with no significant differences: at 3 h, 16.65 ± 7.79 mmHg (DMEK) vs. 16.51 ± 4.65 mmHg (triple-DMEK), *p* = 0.92; at 24 h, 14.73 ± 5.29 mmHg vs. 13.58 ± 2.57 mmHg, *p* = 0.21; and at 48 h, 13.10 ± 3.85 mmHg vs. 12.74 ± 2.90 mmHg, *p* = 0.62. The need for venting was low in both groups, occurring in 4 patients (5%) in the DMEK-alone group and in none of the triple-DMEK patients (*p* = 0.13). Similarly, acetazolamide was used in 6 patients (8.1%) in the DMEK group compared to 1 patient (2.4%) in the triple DMEK group (*p* = 0.16).

### 3.4. Glaucoma vs. Non-Glaucoma

The mean preoperative IOP was 14.2 ± 4.0 mmHg with 2.2 ± 1.6 IOP-lowering medications in glaucoma patients and 13.7 ± 2.5 mmHg in non-glaucoma patients. Five of the 14 patients with glaucoma had undergone a total of nine IOP-lowering surgeries in the past (four trabeculectomies, one SLT, one cyclophotocoagulation, one Hydrus stent, one iStent, and one Ahmed valve). Mean age of the patients and graft size were not significantly different between glaucoma and non-glaucoma groups. All surgeries were performed by two experienced surgeons (JWP and MA), with a distribution of 90.9% versus 9.1% in the non-glaucoma group and 80% versus 20% in the glaucoma group.

When comparing eyes with preexisting glaucoma (n = 16) to those without glaucoma (n = 100), several significant differences were observed in postoperative outcomes. The frequency of venting was low in both groups—3% in the non-glaucoma group and 6.25% in the glaucoma group—the difference was not statistically significant (*p* = 0.51). However, acetazolamide use was significantly more frequent among glaucoma patients (31%) compared to those without glaucoma (3%), *p* = 0.001, indicating a greater need for pharmacologic IOP management in this group. IOP was also significantly higher in the glaucoma group at later time points: at 3 h, IOP was 16.87 ± 6.86 mmHg (no glaucoma) vs. 15.00 ± 6.59 mmHg (glaucoma), *p* = 0.33; at 24 h, 13.92 ± 3.77 mmHg vs. 17.07 ± 7.40 mmHg, *p* = 0.012; and at 48 h, 12.65 ± 3.33 mmHg vs. 14.86 ± 4.13 mmHg, *p* = 0.029. In terms of gas fill, glaucoma patients had significantly lower fill at 3 h (55 ± 17%) compared to those without glaucoma (64 ± 11%), *p* = 0.011, although differences at 24 and 48 h were not statistically significant: 60 ± 15% vs. 55 ± 18% (*p* = 0.26) and 56 ± 15% vs. 50% ± 18 (*p* = 0.24), respectively. These findings suggest that patients with preexisting glaucoma may experience higher postoperative IOP, require more frequent IOP-lowering intervention.

## 4. Discussion

Successful DMEK outcomes rely on a graft with high-quality and high-density endothelial cells, a smooth and precise surgical procedure with minimal manipulation, and adequate postoperative management. IOP elevation in the early postoperative phase is one of the graft- and vision-threatening complications [16]. The most common mechanisms include pupillary block, excessive gas fill, steroid response, and preexisting outflow impairment, particularly in eyes with glaucoma or glaucoma surgery. Ophthalmic surgeons apply different strategies to prevent and manage this issue [17]. In this retrospective study, we analyzed our standardized approach, which includes a surgical iridectomy at the 6 o’clock position, the use of 10% SF_6_ as an endotamponade, and a low-normal intraocular pressure of approximately 15 mmHg at the end of surgery. Furthermore, all patients undergo their first postoperative assessment—including IOP measurement, gas fill evaluation, and iridectomy patency check—as early as three hours after surgery, followed by additional evaluations on the first and second postoperative day.

Intraocular pressure elevation after DMEK, defined as an IOP > 24 mmHg or an increase of ≥10 mmHg from baseline, has been reported in 0% to 24% of patients during the follow-up period [18,19]. Apart from corticosteroid-induced IOP elevation, which typically occurs later, angle closure or pupillary block are the main causes of IOP elevation in the immediate postoperative period. Maier et al. reported postoperative IOP elevation in 15.4% of patients [20]. In their study, preoperatively elevated IOP was the only significant risk factor for postoperative IOP elevation. We observed a stable mean IOP in mid-normal values at the 3 h follow-up in both DMEK and triple-DMEK patients, with a smooth decline in IOP over the first two postoperative days. No cases of pupillary block or angle closure were noted. IOP was slightly higher in glaucoma patients compared to non-glaucoma patients on the first and second postoperative days, but not at the 3 h follow-up. This may be attributed to the perioperative interruption of IOP-lowering medication or to previous glaucoma surgeries, which can alter the geometry of the anterior chamber and the dynamics of aqueous humor outflow. Maier et al. reported preexisting glaucoma to elevate the risk of post-DMEK IOP elevation [10].

The mean gas fill was 63% at the 3 h follow-up and gradually declined to 55% by the second postoperative day. Similarly to IOP, no differences were observed between DMEK and triple-DMEK patients. Gas fill was slightly lower in the glaucoma group compared to the non-glaucoma group at the 3 h follow-up, but this difference was not significant on the following two postoperative days. The lower gas fill observed in glaucoma patients could be attributed to prior glaucoma surgeries, such as trabeculectomy, stents, or tube implants, which may promote faster evacuation of gas from the anterior chamber.

The primary outcome of our study was the frequency and type of IOP-lowering procedures following DMEK. As this was not a prospective trial but a real-life observation, the decision regarding the necessity and type of IOP-lowering intervention was made by the resident on duty—particularly during the 3 h follow-up in the afternoon. According to this assessment, IOP-lowering interventions were performed in 11 eyes (9.5% of the total cohort) during the 3 or 24 h follow-up. Venting only was performed in three eyes, systemic acetazolamide was administered in seven eyes, and one eye received both treatments.

From a clinical perspective, the decision to perform venting in patients #1, #2, #3 and #4 was justified, given the combination of elevated IOP and high gas fill. In contrast, the use of acetazolamide in patients #5 to #11 was based on a “soft” indication and could likely have been managed without any intervention and without negative consequences for the patients. On the other hand, a certain level of awareness—especially among glaucoma patients—is recommended, and it is not a mistake to act with some caution. Nevertheless, these patients required more frequent IOP-lowering interventions than non-glaucoma patients. IOP exceeded 21 mmHg, a commonly used threshold for clinically significant IOP elevation in clinical trials, in only six eyes at the 3 h follow-up, which underscores the conservative nature of our real-world approach. In contrast to other reports, we did not find triple DMEK as a significant risk factor for intervention after DMEK [16].

The fear of releasing all the gas from the anterior chamber and causing DMEK graft detachment should not prevent, especially younger residents, from responding promptly in cases of pupillary block, which poses a threat not only to the graft but also to the optic nerve—not to mention the significant discomfort experienced by the patient. In such cases, venting is the fastest option for lowering intraocular pressure, regardless of whether the gas is located in the anterior or posterior chamber. This method is incomparably more effective than lowering the pressure with systemic or topical medications. Likewise, reopening a small or closed iridectomy with a laser takes more time, involves more manipulation, and is not always feasible in the presence of epithelial edema.

In our study, we did not observe any cases of pupillary block or angle closure. Only two patients presented with an IOP of 45–50 mmHg and a gas fill of 95–100%, requiring venting within the first 3 h postoperatively (1.72%). We believe that a combination of surgical iridectomy at the 6 o’clock position, an SF_6_ concentration not exceeding 10% in the endotamponade, and a low-normal IOP at the end of surgery may be key to maintaining stable postoperative IOP after DMEK. Our data support that IOP elevation after DMEK can be effectively controlled within a few hours after surgery [4,21].

Surgical iridectomy has been shown to be more effective than peripheral laser iridotomy in the study by Steindor et al. [22]. Iridotomies are smaller and carry a higher risk of functional closure due to pigment dispersion, fibrin formation, or contact with the intracameral gas bubble. In our study, no cases of pupillary block were observed, as all eyes had a patent surgical peripheral iridectomy. The use of SF_6_ in the endotamponade, which remains in the anterior chamber longer than air, appears to reduce the need for re-bubbling compared to 100% air [12,23]. On the other hand, too high concentration of SF_6_ can lead to a rapid increase in IOP due to its expansion within the first hours. Based on our data, a 10% concentration of SF_6_ provides a sufficient tamponade duration while minimizing the risk of pressure spikes.

Our re-bubbling rate was 10.3%, which is low compared to the rates reported in the literature, typically ranging from 10% to 30% [12,13]. One of these patients required re-bubbling after venting, as there was no residual gas fill as early as 48 h after DMEK.

Limitations of our study include its retrospective design and the absence of standardized criteria guiding the decision and selection of IOP-lowering interventions except for some conditions described in paragraph 2.3. We are aware that, in contrast to prospective clinical trials with strictly defined algorithms, our study is limited by the lack of a clearly defined decision tree. The decision to perform an intervention or not was based on the clinical judgment of different clinicians. However, we believe that the use of standardized criteria—such as defining an IOP increase above 21 mmHg as an intervention threshold—would likely result in an even lower rate of IOP-lowering interventions than observed in this study. A further limitation of the study is the lack of some baseline data, such as pachymetry or endothelial cell density (ECD), as these examinations are not always part of our routine preoperative diagnostics. Furthermore, postoperative corneal edema may have influenced Goldmann applanation tonometry (GAT) measurements.

## 5. Conclusions

Each patient may respond differently to surgery, and individual risk factors for complications—such as pupillary block—can vary. To prevent severe IOP elevation or pupillary block early after DMEK, standardized surgical technique and meticulous postoperative management are essential. In our standardized approach, which includes a surgical iridectomy at the 6 o’clock position, the use of 10% SF_6_ gas, and ensuring a mid-normal IOP at the end of surgery, no cases of pupillary block occurred in 116 consecutive DMEK procedures. Only two eyes exhibited both elevated IOP and high gas fill at the 3 h follow-up, necessitating a reduction in the endotamponade via venting, which supports the safety and effectiveness of our surgical technique. We believe that our results could serve as a starting point for conducting a prospective clinical study.

## Figures and Tables

**Table 1:** Characteristics of eyes that received intraocular pressure (IOP)-lowering interventions.

	IOP-Lowering Intervention	Age [Years]/Sex	Diagnosis	Comorbidities	3 h IOP/Gas Fill	24 h IOP/Gas Fill	48 h IOP/Gas Fill
**1.**	**Venting at 3 h**	74/F	endothelial decompensation following herpes endotheliitis	vitreous prolapse after complicated cataract surgery	**45/95%**	20/5%	28/0%
**2.**	**Venting at 3 h**	74/M	FECD	none	**50/100%**	12/20%	10%
**3.**	**Venting at 24 h**	65/M	FECD	none	**12/95%**	25/100%	9/60%
**4.**	**Venting + 250 mg acetazolamide p.o. at 24 h**	86/M	FECD	glaucoma	**31/90%**	30/90%	15/60%
**5.**	**250 mg acetazol-amide p.o. at 24 h**	83/F	FECD	glaucoma	**15/60%**	30/60%	14/60%
**6.**	**125 mg acetazol-amide p.o. at 24 h**	81/M	FECD	none	**25/60%**	12/60%	13/50%
**7.**	**250 mg acetazol-amide p.o. + Azopt^®^ at 3 h**	82/M	FECD	glaucoma	**20/65%**	10/60%	12/50%
**8.**	**250 mg acetazol-amide i.v. at 3 h**	85/M	FECD	none	**30/85%**	12/80%	12/90%
**9.**	**250 mg acetazol-amide p.o. + Cosopt^®^ at 24 h**	77/M	FECD	none	**20/65%**	28/65%	16/70%
**10.**	**250 mg acetazolamide p.o. + Cosopt^®^ at 24 h**	80/M	Endothelial decompensation	pseudoexfoliative glaucoma, past glaucoma surgeries	**15/60%**	30/45%	20/40%
**11.**	**500 mg acetazolamide p.o. + Cosopt** **^®^ at 3 h**	70/M	FECD	glaucoma, past trabeculectomy	**34/60%**	24/60%	19/50%

FECD—Fuchs’ endothelial corneal dystrophy, F—female, M—male, h—hour, p.o.—per os, i.v.—intravenous.

## Data Availability

Data available on request due to legal restrictions.
